# Transient cavity dynamics and divergence from the Stokes–Einstein equation in organic aerosol†

**DOI:** 10.1039/c9sc06228a

**Published:** 2020-02-17

**Authors:** Young-Chul Song, Stephen Ingram, Robert E. Arbon, David O. Topping, David R. Glowacki, Jonathan P. Reid

**Affiliations:** School of Chemistry, University of Bristol Cantock's Close Bristol BS8 1TS UK j.p.reid@bristol.ac.uk glowacki@bristol.ac.uk; Centre for Computational Chemistry, University of Bristol Cantock's Close BS8 1TS UK; School of Earth and Environmental Science, University of Manchester Manchester M13 9PL UK; Department of Computer Science, University of Bristol UK

## Abstract

The diffusion of small molecules through viscous matrices formed by large organic molecules is important across a range of domains, including pharmaceutical science, materials chemistry, and atmospheric science, impacting on, for example, the formation of amorphous and crystalline phases. Here we report significant breakdowns in the Stokes–Einstein (SE) equation from measurements of the diffusion of water (spanning 5 decades) and viscosity (spanning 12 decades) in saccharide aerosol droplets. Molecular dynamics simulations show water diffusion is not continuous, but proceeds by discrete hops between transient cavities that arise and dissipate as a result of dynamical fluctuations within the saccharide lattice. The ratio of transient cavity volume to solvent volume increases with size of molecules making up the lattice, increasing divergence from SE predictions. This improved mechanistic understanding of diffusion in viscous matrices explains, for example, why organic compounds equilibrate according to SE predictions and water equilibrates more rapidly in aerosols.

## Abstract 2

The failure of the Stokes–Einstein relation is assessed in aerosol measurements and molecular dynamics simulations.

## Graphical abstract

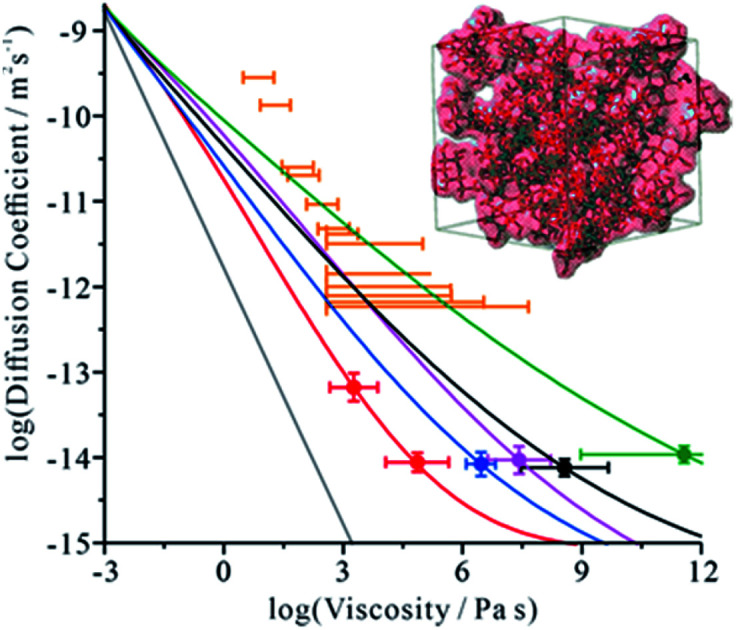

## 1. Introduction

Examining the relationship between the diffusion rates of small molecules and the viscosity of the surrounding molecular matrix is important for exploring problems as diverse as the molecular mechanisms of crystallization and the formation of amorphous phases in drying droplets,^1–3^ the controlled-release of active ingredients from structured micro-particles in pharmaceutical and consumer products,^4–7^ and the mass concentration of secondary organic aerosol particles in a polluted urban environment.^8–10^ The simplest relationship, the Stokes–Einstein (S–E) equation, expresses the inverse correlation between the translational diffusion coefficient, *D*, of a large spherical solute molecule of radius *a*, moving within a solvent continuum with a dynamic viscosity, *η*:^11,12^1
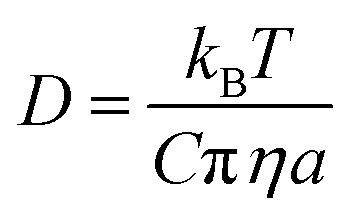
where *C* is a constant. However, in many important cases the “solvent” (*i.e.* the dominant component by mole fraction) may be a large organic molecule and the “solute” (*i.e.* the minor component) may be a small molecule, *e.g.* water.^11,13,14^ For example, in the drying of aqueous-organic solution droplets, the evaporation of water can lead to an involatile solute surpassing its solubility limit, thereby becoming the major component with a mole fraction that can approach 1. The sudden removal of water can lead to a “frozen” organic-rich matrix with a sufficiently high viscosity such that nucleation and crystallization are delayed, unable to occur on an experimentally realisable timescale, with the solution composition crossing the threshold for a moisture-induced glass transition.^15^ Even then, the residual moisture content can impact product lifetime and particle morphology. Under these conditions, it is most appropriate to consider the diffusion of water within an organic matrix at infinite dilution of water; however, it is typical that a significant divergence from the S–E equation is observed in this limit.^16–18^ Modifications to the S–E equation have been suggested, including the use of a fractional exponent (*i.e. D* ∝ *η*^−*α*^), that account for different relationships between the diffusion coefficient and viscosity.^19–21^

Independent measurements of diffusion coefficients and viscosities over the appropriately wide ranges needed to observe the failure of the S–E equation are challenging. Most measurements report the *temperature-dependence* of viscosities and diffusion coefficients for super-cooled liquids or solutions of fixed composition, and can approach close to the glass transition temperature.^12,22–24^ By contrast, there are many fewer studies of the *compositional dependence* of the divergence from the S–E equation, for example with diminishing moisture content as the glass transition relative humidity (RH) is approached.^11,13,17,18,20,25^ Moisture acts as a plasticizer in atmospheric aerosol particles, regulates the viscosity and, thus, shelf-life of amorphous particles used in formulations, and could play a critical influence in governing crystal formation in drying droplets and films as opposed to the formation of an amorphous solid. Examining the compositionally dependent divergence of an organic solute–water mixture from S–E behaviour not only requires accurate measurements of diffusion coefficients and viscosities over as much as 15 orders of magnitude but requires accurate measurements of composition, recognising that both viscosity and diffusion coefficients are highly dependent on the identity for the functional groups forming the organic solute.^26^ To access the full viscosity range, moisture must be removed from metastable supersaturated solution droplets without crystallization. We report here a systematic study of the failure of the S–E equation for a range of aqueous-saccharide solutions, varying the molecular size of the organic molecule forming the viscous matrix relative to water and exploring the detailed mechanism of water transport in the limit of a pure saccharide particle.

## 2. Measurements of diffusion coefficients of water in aqueous-saccharide aerosol particles

Not only are saccharides used widely as excipients for drug delivery^27–29^ and excipient particles are often prepared by spray drying,^30–32^ they find widespread application in the food industry and are commonly used as laboratory surrogates for high oxidized viscous secondary organic atmospheric aerosol.^3,15,33–36^ Using aerosol particles levitated in optical tweezers, we have carried out measurements which avoid the process of heterogeneous nucleation that occurs in the presence of a substrate, allowing access to particle viscosities spanning dilute aqueous solutions (10^−3^ Pa s) to an amorphous solid (10^12^ Pa s). The moisture content is readily altered by varying the relative humidity of the gas phase. Specifically, we considered five binary aqueous-saccharide solution aerosols: glucose (a mono-saccharide); sucrose, trehalose and maltose (all di-saccharides); and raffinose (a tri-saccharide). We also consider aqueous aerosol droplets containing levoglucosan, a representative oxygenated compound of biomass burning aerosol particles in the atmosphere.^37^

**Fig. 1:**
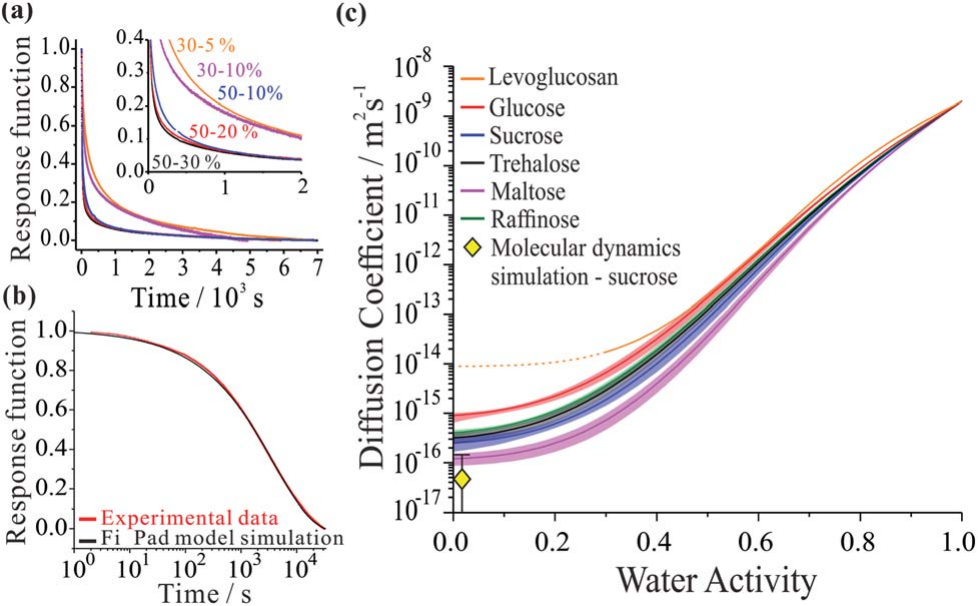
Examples of each step in the workflow required to extract the compositional dependencies the diffusion coefficients from a time-dependence in particle size. The panels show: (a) a collection of response functions for size changes of aqueous-raffinose particles following a step change in RH; (b) a single response function following a change in RH from 30 to 5% RH for a sucrose particle and the best-fit produced by the Fickian diffusion model. (c) The estimated compositional dependencies of the diffusion of water in the six binary aqueous-organic aerosol systems studied. The estimate of the diffusion coefficient for water in sucrose from the MD simulations also presented (yellow diamond).

Fig. 1a shows examples of the time-dependence in the size response functions for aqueous-raffinose particles following transitions in RH. The significant changes in the equilibration time reflect the significant changes in particle viscosity that are observed over this range in RH/moisture content: equilibration at RHs above the glass-transition RH occurs on timescales ≪1 hour; at low RH, the release of moisture from an amorphous glass occurs over many hours and indeed is not complete over the experimental timescales. The time-constant, *τ*, and “stretch factor” *β* of the multiexponential decay observed in both evaporation and condensation events show a path dependence, varying with both the initial and final RH, the initial particle size and the wait-time at intermediate RHs (see Fig. S4†). Tabulated values of both parameters observed in each of the new systems may be found in Table S1.† To fit the compositional/water activity dependence of the diffusion coefficient of water for binary solution aerosol droplets requires measurements at many RH transitions.^38,39^

Measurements were performed over 6 RH transitions for 96 droplets for the six binary aqueous-organic aerosol systems studied (glucose, sucrose, trehalose, maltose, raffinose and levoglucosan). Transitions in size were slowest for maltose droplets at the lowest RHs. Moreover, the characteristic timescale increases with increasing particle size for every binary organic system studied (see Fig. S3 and Table S1†). Time-constants for all particle sizes in the range 3–6 μm show the same ordering: maltose > raffinose > trehalose > sucrose > glucose ≥ levoglucosan. In other words, the chain length of the organic appears to be important to the internal mixing dynamics but is not the only controlling factor.

The compositional dependencies of the diffusion coefficients of water estimated for these binary aqueous-organic systems are summarized in panel (c). For reference, the moisture driven glass transition RH has been reported as 53% for raffinose^36,40^ and 32% for maltose;^36^ the majority of evaporation measurements for these two systems have been made with ultra-viscous and even glassy particles. A value of 23% RH has been reported for sucrose^36,40^ while glucose and levoglucosan are not expected to become glassy at any moisture content at this temperature;^34^ indeed, levoglucosan crystalizes at an RH of 30% and diffusion coefficients cannot be measured below this. The trend in *D*_w_ is not monotonic with molecular weight: levoglucosan (162.1 g mol^−1^) > glucose (180.2 g mol^−1^) > raffinose (504.4 g mol^−1^) > trehalose (342.3 g mol^−1^) > sucrose (342.3 g mol^−1^) > maltose (342.3 g mol^−1^) at the same water activity. Water in the monosaccharide shows the fastest diffusivity, and diffusion in the trisaccharide is faster than in the disaccharides when a fixed RH/water activity is considered. Indeed, this trend in the diffusion coefficient of water in the limit of a pure dry organic matrix is consistent with a previous assessment of the diffusion coefficients at the glass transition temperature for a subset of the compounds studied here.^41^

## 3. The relationship between diffusion and viscosity in mono-, di- and tri-saccharide particles

**Fig. 2:**
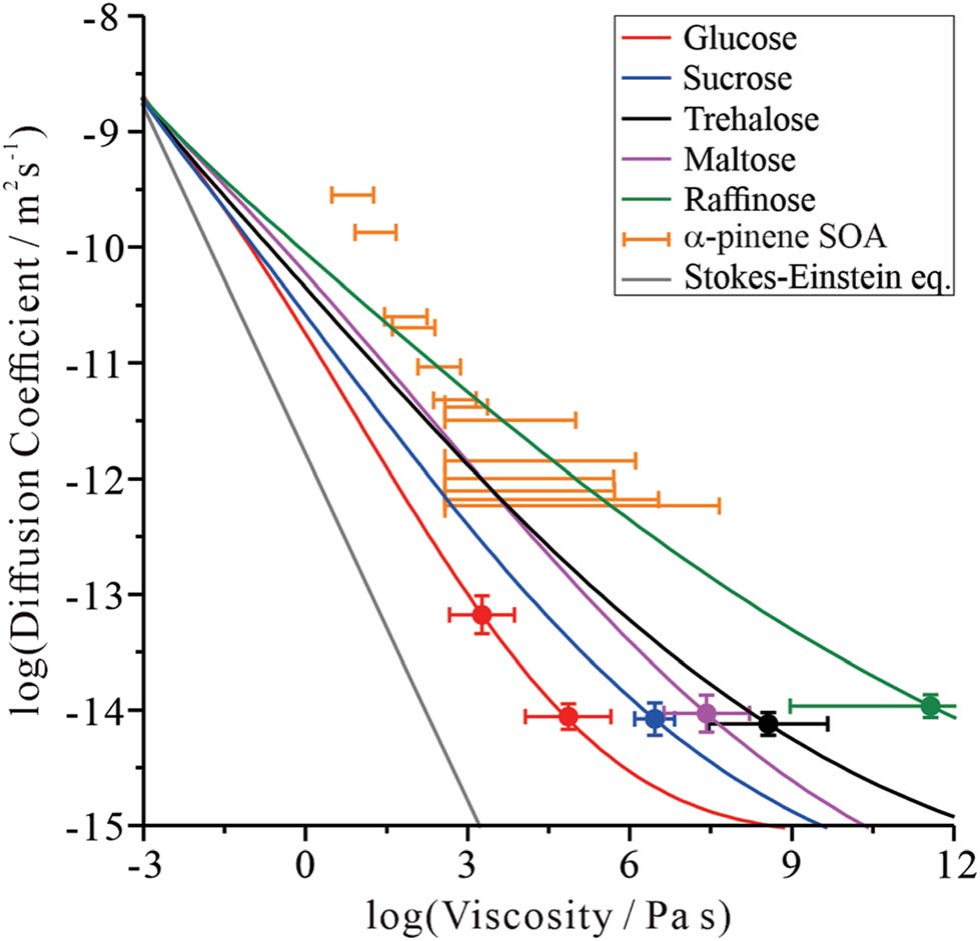
Correlation of the diffusion coefficient of water with the viscosity of the aqueous-organic matrix. A prediction from the S–E equation is shown by the grey line. The relationship between the diffusion constant and viscosity of α-pinene SOA (orange markers) has been inferred by us from the literature, and has previously been discussed.^17,46^ The colour scale is the same as in Fig. 1.

The diffusion coefficient measurements presented in Fig. 1 and our measurements of solution droplet viscosities^36^ allow us to examine their correlation over wide ranges spanning more than 12 orders of magnitude in viscosity and 7 orders of magnitude in diffusion coefficient. The correlations for these systems are compared with predictions from the S–E equation in Fig. 2, assuming a molecular diameter for water of 0.2 nm. Typical error estimates in diffusion coefficient and viscosity are indicated by the representative error bars for each system. The diffusion coefficients for water in all organic-aqueous solutions increasingly deviate from the S–E equation with decreasing water activity and increasing viscosity. Even at the threshold of semi-solid behaviour (10^4^ Pa s), the diffusion coefficient of water in aqueous-raffinose aerosol droplets is ∼5 orders of magnitude larger than estimated by S–E. This is a consequence of the inapplicability of the S–E assumptions to estimations of the diffusion coefficient of a small molecule moving within a matrix of large molecules, *i.e.* the translation of water is not characterized by simple Brownian motion.^23,42^

Comparing the relative divergence of water diffusion coefficients from S–E predictions for the mono-, di- and tri-saccharides, the discrepancy increases systematically across this series. Water transport is fastest in solutions with the tri-saccharide raffinose and slowest in solutions with the mono-saccharide glucose at a certain solution viscosity; the di-saccharides (sucrose, trehalose and maltose) fall in the intermediate range. These results suggest that the disparity in size between water and the organic molecule forming the matrix is key to determining the diffusion rate of water. It also explains why the particle size relaxation times and limiting *D* values (in dry air) did not directly scale with molecular weight: the particles exhibit different viscosities at the same water activity. Therefore, an independent viscosity axis, in this case produced using the aerosol particle coalescence technique, is crucial to separating the two effects.

It can be postulated that when forming a matrix from raffinose, a much larger molecule than water, the packing density of raffinose leaves sufficient free volume for water to move more readily through the network of organic molecules. When the organic molecule is closer in size to water, as in the case of the mono-saccharide glucose, the tighter relative packing of glucose leads to a fewer adequately sized cavities. In this sense, the mechanism of impaired water transport more closely resembles percolation rather than diffusion,^43,44^ a process that is sensitive to the free volume of the medium.^45^

Fig. 2 is instructive when considering the diffusion of water through the complex organic matrices found in atmospheric secondary organic aerosol (SOA), one particular motivation for the current study. For example, water transport in α-pinene SOA (orange bars) is more rapid than would be expected based on measurements of viscosity and estimates from the S–E equation,^11,14,17^ most closely resembling the di- and tri-saccharides. However, it should be recognised that the properties of SOA constituent molecules are considerably different. The average molecular weight of organic components identified in α-pinene SOA is 150–200 g mol^−1^,^47^ albeit with a lower degree of oxygenation: typically the O : C ratio has been reported as 0.45–0.55.^48^ The O : C ratios for trehalose and raffinose are 0.92 and 0.89, respectively. The faster diffusion of water in SOA than expected from the S–E equation may be attributed to the heterogeneity in composition at the molecular scale, leading to a porous network of channels through which water transport is more facile than expected.

## 4. The microscopic mechanism from molecular dynamics simulations

To better understand the microscopic mechanism of water transport, we carried out atomistic molecular dynamics (MD) simulations of water in sucrose (with ‘concentrations’ of one water molecule per 35 sucrose molecules) designed to mimic experimental water activities close to zero. See ESI† for further information on the MD simulations. The initial placement of the organic molecules is intended to replicate the amorphous packing structure that is expected to occur near the surface of a glassy sucrose droplet.^49^ These MD simulations were analysed to provide an independent estimate for the value of the intercept *D*_w,org_. Fig. 1c shows the MD-derived value of 4.64 × 10^−17^ m^2^ s^−1^ is in good agreement with the experimental measurements, and indicates that our computational approach captures the physics of water diffusion in sucrose at low activities.

**Fig. 3:**
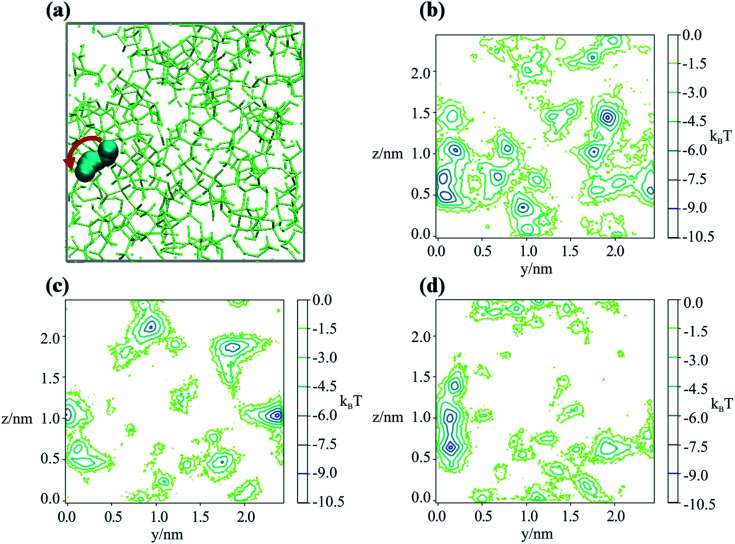
(a) Snapshots in the *yz* plane of the trajectory of a water molecule ‘jumping’ between interstices in an amorphous sucrose lattice. The red arrow indicated the direction of the observed hop. The periodic box is shown in grey. Sequential calculated potentials of mean force that the water experiences are shown in panels (b) to (d), separated in time by 1 ns.

**Fig. 4:**
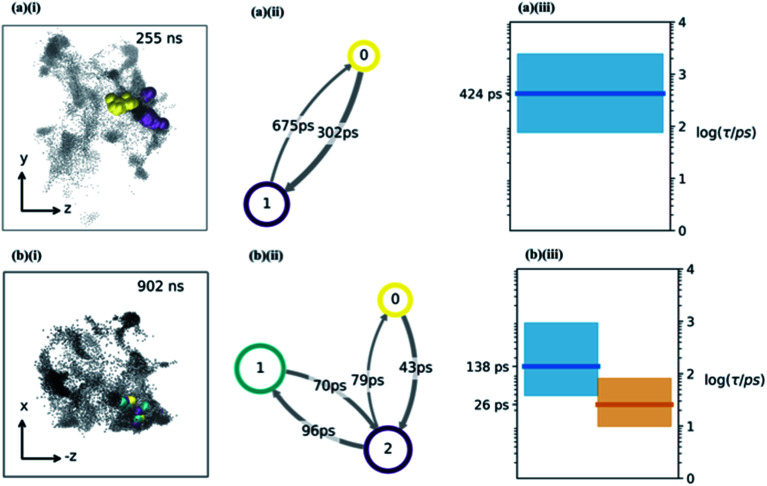
(a and b) The water hopping mechanism showing 2 and 3 metastable states (panels a & b respectively) arising from one of the nine trajectories. Subplots (i) show *zy*, (−*z*)*x* projections of water molecule's position throughout the trajectory with different colours indicating different metastable states. Subplots (ii) shows a hidden Markov state model representation of the hopping behaviour. Each circle represents a metastable state, with the size related to its stability, the arrows show the hopping timescale in picoseconds from one state to another (same colour scheme as (i) subplots). Subplots (iii) show Bayesian estimates of the relaxation timescales associated with hopping between the states (thick line is mean, coloured region is a 95% credibility interval).

Inspection of our MD results reveals that the mechanism of water diffusion through the sucrose matrix proceeds by a hopping between cavities (Fig. 3). In general, a ‘cavity’ is defined as a sucrose interstitial domain where water has a significant lifetime based on Markov analysis. Our analysis has enabled us to identify both reversible and irreversible examples of intercavity dynamics (see ESI†). Fig. 4 and much of the analysis described in this article focusses on clusters of ‘cavities’ between which water molecules make reversible kinetic hops, because this local equilibrium is amenable to analysis using standard tools in statistical mechanics.

Our analysis shows that water remains in a cavity (or cluster of cavities) until either (1) it achieves sufficient kinetic energy to escape the local environment, or (2) the slower dynamics of the sucrose matrix opens a pathway that allows access to a new cavity. This appears similar to the ‘micropore diffusion’ mechanism, which has been proposed to describe the uptake and transport of small molecules through porous zeolite structures.^50^

In order to determine the time-dependent dynamics of the cavities, we identified a 3 ns timeslice of a 1 μs trajectory, where we observed a water molecule jumping between two distinct cavities, as illustrated in Fig. 3a. Over the course of the 3 ns timeslices, we extracted three different equilibrium configurations, and ran a 50 ns MD simulation beginning from each of these points, freezing the sucrose but not the water. The purpose of these simulations was to use the water molecular as a “probe” of the cavity structure and dynamics, in order to understand cavity persistence on the timescale of a typical water hop. The potential of mean force within each cavity (PMF), without the entropic degrees of freedom of the organic matrix included, was determined by Boltzmann weighting the resultant probability distribution, *P*:

2 PMF = −*k*_B_*T* ln(*P*)

Fig. 3b–d shows that there is a small but noticeable change in the cavity PMF landscape (the region around *y* = 0.1, *z* = 0.5–1) as the sucrose reorients over 3 ns. This observation is consistent with analysis showing that the position–position autocorrelation function of a single sucrose molecule decorrelates after approximately 1 ns, as presented in Fig. S8.†

Having determined an approximate upper limit on the sucrose re-organisation timescale, we split each trajectory up into 1 ns slices and determined the kinetic parameters for water-hopping between cavities using a Bayesian Hidden Markov (HM) modelling approach. For each 1 ns slice, we constructed a HM model to determine the number of metastable states (cavities), the relative population of water within each cavity, transition probabilities for hopping between cavities, and the timescales for inter-cavity transport. The HM analysis shows that the cavities have a distribution of free energies (Fig. S9†), and a corresponding distribution of lifetimes for water within any given cavity. Fig. 4a and b show representative examples from three 1 ns slices throughout one MD simulation where water hops between clusters of two and three cavities, along with information regarding the hopping timescales in subplots (ii). Subplots (iii) show the timescales of interstate rearrangement processes. These timescales do not correspond to pairwise hopping between cavities but rather joint relaxation processes over all states.

Extended periods of cavity hopping behaviour are found in all our 300K MD simulations: water repeatedly moves back and forth between adjacent cavities that do not fully collapse once they are vacated. The characteristic barriers for hopping between cavities have been calculated using Transition State Theory and are found to be on average 6.42 (±1.29) *k*_B_*T*. The distribution across all trajectories (Fig. S9†) corresponds to a hop frequency of between 1 and 50 per nanosecond per water molecule, although not all hops will lead to productive diffusion against a concentration gradient. In fact, ‘return trips’ may be a common feature of water transport in these matrices.

**Fig. 5:**
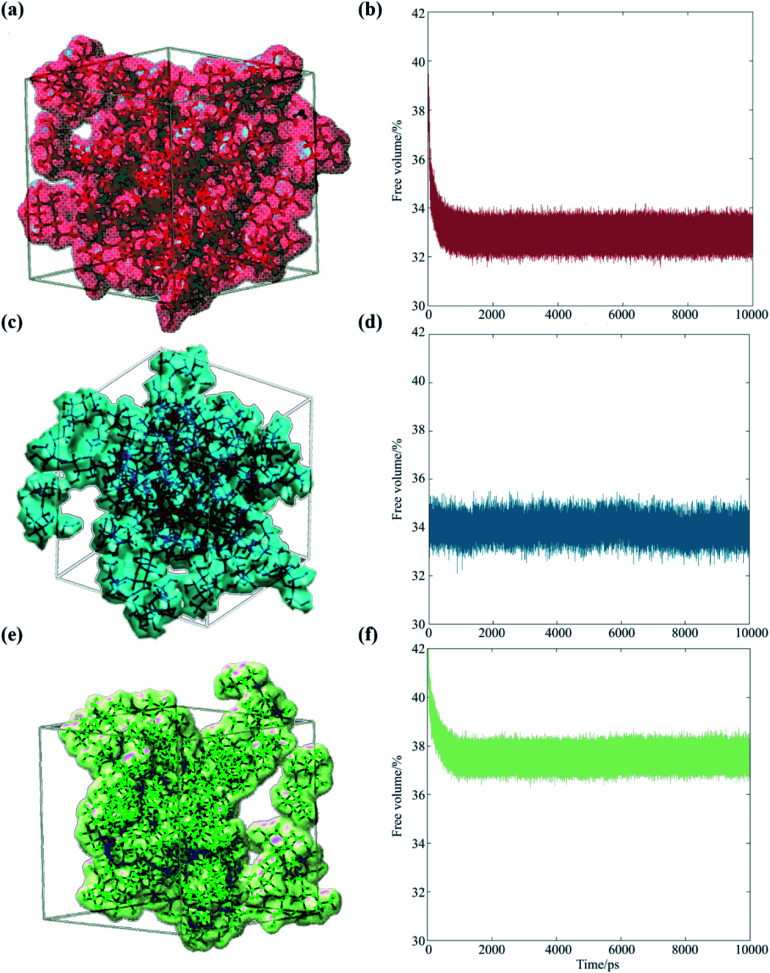
Occupied volumes of (a) glucose (67.1%), (c) sucrose (66.0%) and (e) raffinose (62.4%) matrices are shown (same colour scheme as Fig. 1 again) showing the van der Waals radii of the saccharides within one snapshot of the short simulations. Fractional free volume are shown for (b) glucose, (d) sucrose, and (f) raffinose calculated by GROMACS throughout 10 ns simulations.

Our analysis suggests that the magnitude of the S–E deviation depends on the transient packing efficiency of the organic molecules. For instance, raffinose self-diffuses slower than sucrose (hence the observed particle viscosity is higher), but if the average volume of cavity space within the lattice is larger and more highly connected, then the net water flux will be higher at a given particle viscosity. To evaluate this, we carried out a series of MD simulations, which we post-analyzed to assess the cavity volume within glucose, sucrose and raffinose matrices. The final coordinates of the three matrices are presented in Fig. 5, showing increasing cavity size and density within the van der Waals surfaces. We also express this quantity as a fraction of the simulation volume in Fig. 5.

Thus, the trend in viscosity data (Fig. 2) can be rationalised at a molecular level: the ‘hopping’ mechanism of water transport will become more efficient as the size of the organic constituent increases. Therefore, the frictional forces experienced by water molecules will deviate further from those assumed by eqn (1), and the observed *D* will be under-predicted to a greater extent for larger organics. With reference to atmospheric organic aerosol, this effect may be significant in particles containing large numbers of oligomeric or ‘humic-like’ molecules. Such constituents are frequently found in aerosol formed under low RH,^51^ low temperature^52^ or high precursor concentration^53^ conditions.

## 5. Conclusion

In conclusion, we have shown that the diffusion constant of water in viscous aerosol particles departs increasingly from the SE equation as the size of the saccharide molecule forming the matrix increases. Atomistic simulations suggest that larger molecules will pack less efficiently, facilitating a mechanism of activated hopping through the porous network: at high saccharide fraction, a water molecule executes discrete jumps between cavities at a rate governed by the collective motion of the saccharide matrix. These observations also are consistent with the slower diffusion of molecules larger than water, whose motion more closely resembles that described by Stokes flow.^24^

## Conflicts of interest

There are no conflicts to declare.

## Supplementary Material

SC-011-C9SC06228A-s001
