# Supplementary material for: Transient cavity dynamics and divergence from the Stokes–Einstein equation in organic aerosol
Source: Chem Sci. 2020 Feb 17;11(11):2999–3006. doi: 10.1039/c9sc06228a (PMC8157714; doi:10.1039/c9sc06228a)
Supplement: SC-011-C9SC06228A-s001 [file SC-011-C9SC06228A-s001.pdf]

Electronic Supplementary Material (ESI) for Chemical Science.
This journal is © The Royal Society of Chemistry 2020

Supporting Information for:

Transient Cavity Dynamics and Divergence from the Stokes-Einstein Equation in Organic Aerosol

Young-Chul Song,[1] Stephen Ingram,[1,2] Robert Arbon,[1,2]
David O. Topping,[3] David R. Glowacki,[1,2,4,*] Jonathan P. Reid[1,*]

[1] School of Chemistry, University of Bristol, Cantock's Close, Bristol BS8 1TS
[2] Centre for Computational Chemistry, University of Bristol, Cantock's Close BS8 1TS
[3] School of Earth and Environmental Science, University of Manchester, Manchester M13 9PL
[4] Department of Computer Science, University of Bristol
Correspondence to: Jonathan P. Reid (j.p.reid@bristol.ac.uk) and David R. Glowacki (glowacki@bristol.ac.uk)

Keywords: Aerosols, viscosity, diffusion coefficient

The Experimental Procedure for Determining Timescales of Water Transport

In order to determine evaporation and condensation timescales for binary systems that are mixtures of water and organic compounds, they were subjected to a single transition step of relative humidity (RH) across different particle size and between different pair of target RH:

An individual binary solution aerosol droplet (3-10 μm radius) is captured at high RH/dilute saccharide concentration in a gradient-force optical trap (optical tweezers) formed by a tightly focused laser beam (wavelength 532 nm)[1,2]. After a period of conditioning at an elevated RH (typically 70-80 %), the mixing ratios of humidified and dry nitrogen are altered to drive a change in RH in the trapping cell. Measurements proceed either through small downward (or upward) steps in RH of 10% or following large changes of 30% or more. The moisture content of the droplet responds accordingly, attempting to remain at a water activity equal to the surrounding RH, driving a change in particle size through the evaporation or condensation of water.

The time-dependence in the particle size and refractive index (hence composition and moisture content) are inferred (with sub-nm precision for size) from the shifts in the wavelengths of whispering gallery modes apparent on the Raman O-H stretching band of water [3–5].

Figure S1 shows one example of KWW fitting for the aqueous-sucrose system. The response in particle size is inferred from the wavelength shift indicated by the red line, starting at a particle size of 3650 nm and a wavelength of 655.4 nm following an initial period of 3 hours conditioning at 30% RH. From this point, the KWW equation was applied to fit the evaporation step (RH 30% to 5%) as shown by the blue line. The fitting of the condensation step (RH 5% to 30%) is shown by the green line. The timescales of water transport for each process were 1841 s and 2566 s, respectively. As the fitting results demonstrate, the KWW equation is a suitable method to determine the relaxation process.

It has previously been shown that stretched exponential relaxation reflects the departure from a single-exponential relaxation in size as the particle becomes increasingly viscous [6],

$$F(t) = \frac{r(t) - r(t = \infty)}{r(t = 0) - r(t = \infty)} = \exp\left[-\left(\frac{t}{\tau}\right)^{\beta}\right], \qquad (1)$$

where t is the experimental time, τ is the timescale of relaxation and β characterizes the shape of the multi-exponential time-dependence. The response function, $F(t)$, characterizes the fractional relaxation from the initial particle radius r at t=0 s through to the final equilibrated radius at t=∞ following the change in RH.

This KWW fitting process has been carried out using customized LabVIEW software.

Figure S1. On the binary-sucrose system, RH transition is from 30% to 5% for the evaporation step, and 5% to 30% for the condensation step. The black line is RH profile and the red line is one of WGMs change. The blue and green lines are fitted by KWW equation (eq. 1). The timescales of evaporation and condensation are 1841 s and 2566 s, respectively.

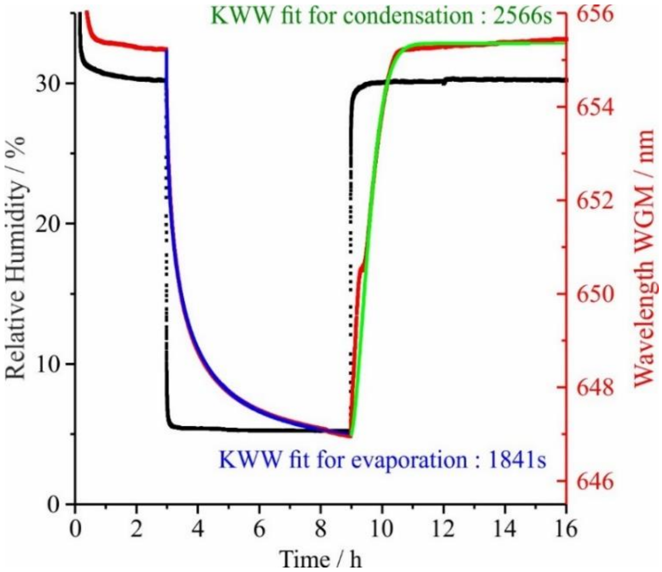

The Effect of Particle Size on Equilibration Time (τ) for Six Binary Organic Systems

Before moving on to fit the compositional dependence of diffusion constants, we consider first the qualitative dependence on droplet size. The experiment conditions are divided into two main categories for evaporation and condensation in this study: a low viscosity transition (RH 50% to 30% and RH 30% to 50%) and a high viscosity transition (RH 30% to 5% and RH 5% to 30%). The six panels in Figure S2 show measurements for six binary systems of aqueous-glucose, aqueous-sucrose, aqueous-trehalose, aqueous-raffinose, aqueous-maltose, and aqueous-levoglucosan. All measurements for the six binary organic mixtures clearly show different trends with respect to initial or final RH, but the characteristic timescale of water transport (τ) clearly also shows a particle size dependence during evaporation or condensation.

In the high relative humidity region, transitions RH 50% - 30% - 50% (i.e. the evaporation step of RH 50% to 30% and condensation step of RH 30% to 50%), the droplets of maltose showed the largest value of timescale of water transport (τ) over the other organic particles, see Figure S3. Moreover, the characteristic timescale increases with increasing particle size for every binary organic system. For example, the characteristic timescale of raffinose is 188s for 3332nm, 265s for 4110nm, and 313s for 4924nm. Comparing similarly sized particles of ~4 μm during an evaporation step, the organic particles show 514s ± 31s for maltose (4114nm), 250s ± 23s for raffinose (3967nm), 157s ± 13s for trehalose (4101nm), 59s ± 19s for sucrose (4016nm), 29s ± 3s for glucose (3871nm), and 32s ± 4s for levoglucosan (3746nm). The ordering of the timescales for all particles sizes in the range of 3 - 6 μm show the same tendency: maltose > raffinose > trehalose > sucrose > glucose ≥ levoglucosan, as shown in Figure S3. In this region, raffinose and maltose particles have experienced the glass transition at RH ~53% and RH ~32% in the ambient temperature.[2,7] However, trehalose, sucrose,

glucose and levoglucosan do not pass through the glass transition RH until a much lower value. The molecular diffusivity of water in each organic system will be treated in the next section.

Figure S2. Examples of the response function for size changes of six binary mixtures particles following a step change in RH

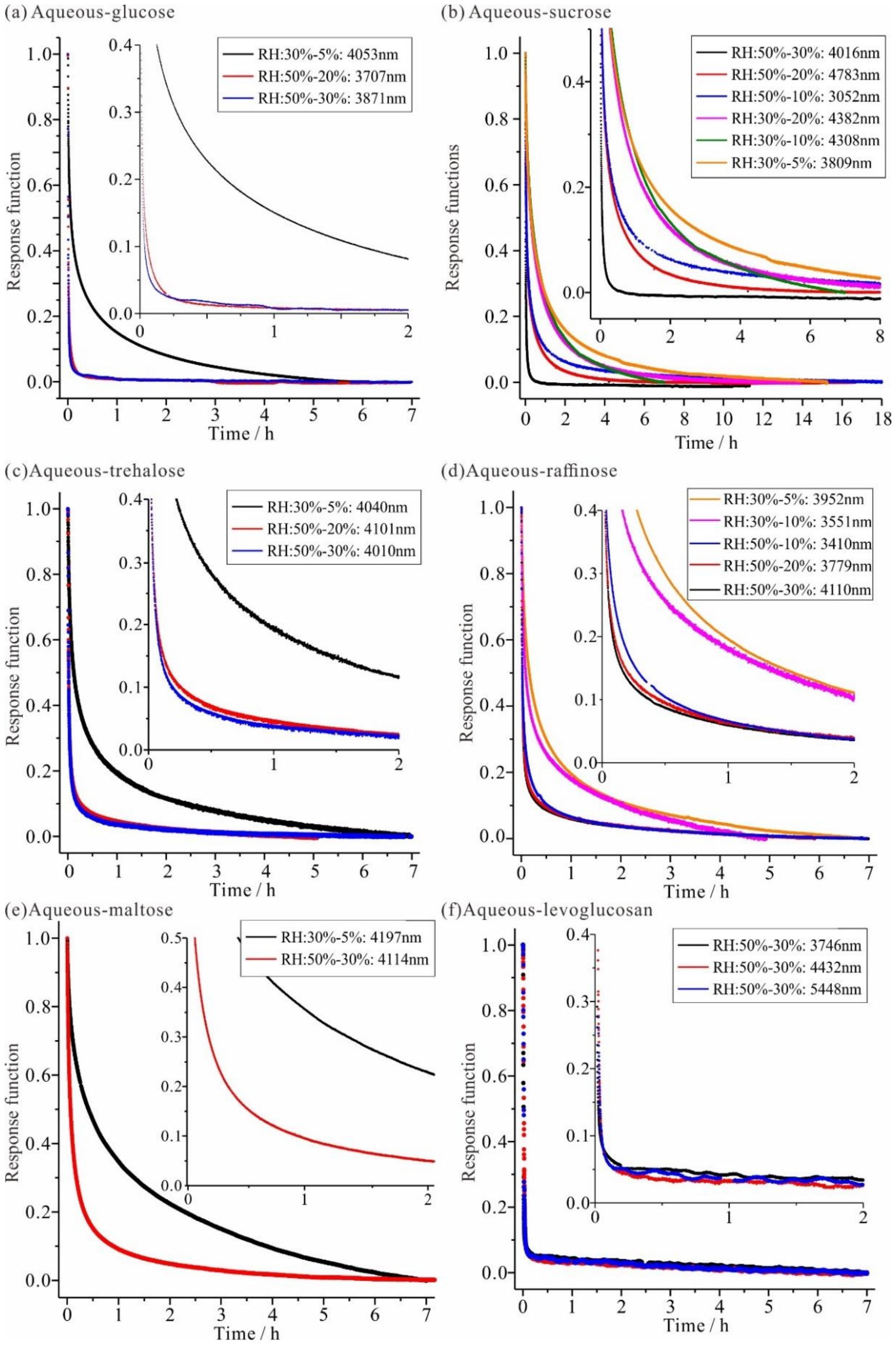

Figure S3. Timescale of water transport (τ) for evaporation step and condensation steps (reported numerically in Table S1). The evaporation and condensation timescales are determined by KWW function, and particle size is calculated from the droplet Raman signal by the proprietary LARA software. Compositions are: (a) binary aqueous-glucose (b) binary aqueous-sucrose (c) binary aqueous-trehalose (d) binary aqueous-raffinose (e) binary aqueous-maltose (f) binary aqueous-levoglucosan

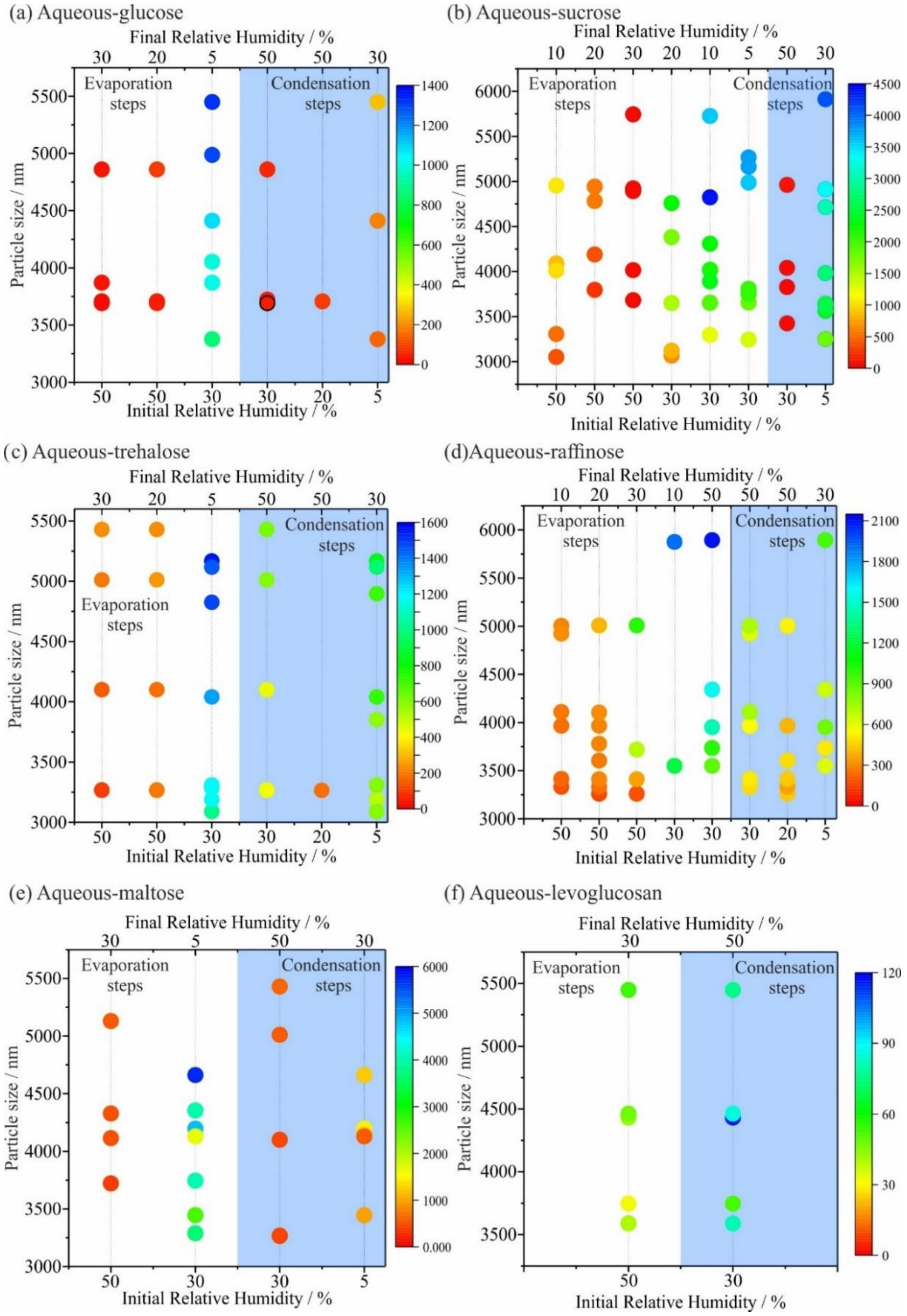

For transitions at low relative humidity, notably for RH 30% to 5% and 5% to 30%, which is the high viscosity region for all compounds, the characteristic timescales of water transport of maltose particles have the longest water transport timescales in the low humidity region. Comparing similar sized particles of ~4 μm during an evaporation step, the organic particles showed the timescales as follows: 4062s ± 55s for maltose (3745nm), 2035s ± 23s for sucrose (3809nm), 1447s ± 19s for raffinose (3952nm), 1342s ± 17s for trehalose (4040nm), and 1009s ± 10s for glucose (3871nm). Levoglucosan cannot be measured in this RH region with particles crystallising at higher RH. The ordering of the timescales for all particles sized in the range of 3 - 6 μm show the same tendency; maltose > sucrose > raffinose > trehalose > glucose, as shown in Figure S2. In this low RH region, raffinose (RH ~53%)[2,7], sucrose (RH ~23%)[2,7], trehalose (RH ~22%)[2,8], and maltose (RH ~32%)[2,9] pass through the glass transition RH at ambient temperature except glucose, and glucose shows the smallest timescale for water transport. The characteristic timescale of water transport can be explained by water diffusion in the organic particles. All water transport experimental data go into Fi-PaD diffusion simulation.

Equilibration Time (τ) Variation with "Wait Time" Effect

The relaxation dynamics for condensation processes are dependent on the "wait time" (i.e. also referred to the aging of the particle). Figure S4 shows the impact of "wait time" on the relaxation timescale of water transport in a binary water-sucrose system. In this case the relaxation timescale is the timescale for the re-condensation of water following a period of drying of varying time (the "wait time". Three different experimental conditions were studied. Sucrose particles that are indicated in black squares were dried for 5 hours at RH 30%, and then the particles were held at RH 5% for 6 hours. For comparison with this data set, sucrose droplets, indicated by red circles and blue triangles, were held at 12 and 24 hours at RH 5%. After 6, 12, and 24 hours at 5% RH, the RH is restored to the initial level 30% RH. Several sucrose particles across a range of particle size (3 - 5 μm) were studied at each RH transition. Figure S4 shows that when the particles experienced a long wait time, the relaxation timescale of water transport (τ) increased during re-condensation following the increase in RH to the initial level. Depending on wait time under dry conditions, the water content varies both in magnitude and spatially before the RH is increased.[6] When increasing RH again, water vapor immediately condenses onto the particle surface, and leads to a unique level of heterogeneity which varies with the timescale over which moisture has been removed and the particle dried.[6,10] The KWW equation was used to determine the timescales for the re-condensation step and the dependence on particle size is shown in the Figure. When the particle size is larger, the timescale for re-condensation is also greater. The three trend lines in the Figure, fitted by a linear equation, clearly indicate the relation between drying time, particle size and re-condensation timescale. The significance of the wait time is that when particle returned to the initial state from dry condition, the condensation time to return the particle to equilibrium with the surroundings increases as the wait time increases.

Figure S4. Timescale of water transport (τ) for each condensation step of sucrose, RH change from 30% to 5% then back to 30% after drying. Particles of black squares experienced 6 hours drying time at RH 5%, Particles of red circles experienced 12 hours drying time at RH 5%, and Particles of blue triangles experienced 24 hours drying time at RH 5%. Error bars were calculated a variation of β ± 0.1.

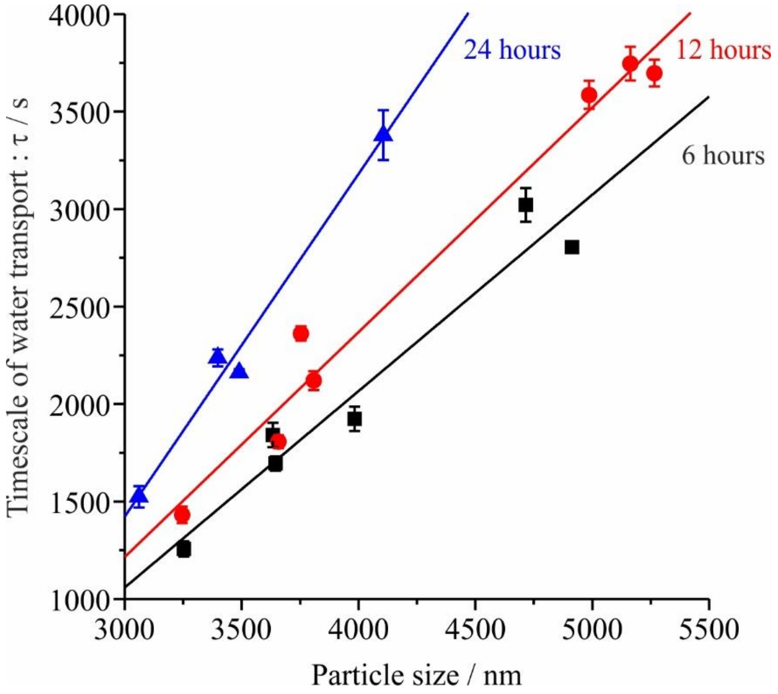

Fickian Diffusion Modelling (Fi_PaD model) for Determining Diffusivity of Water in Aerosol Particles

In order to determine diffusivity of water in organic mixtures, a recently developed Fi_PaD model is applied to experimental data in this thesis. This was achieved using the same WGMs as were selected to fit KWW functions to.

O'Meara *et al*. developed a partial differential model, called Fi_PaD model, comparing it with two other diffusion models referred to as the ETH model and KM-GAP model (kinetic multi-layer model of gas-particle interactions in aerosol and clouds).[11] The Fi_PaD model is established using Fick's second law. The Fi_PaD model uses three initial assumptions below:

1. An aerosol particle is spherical.
2. A spherical particle is divided into inner concentric shells.
3. A surface shell immediately reaches an equilibrium state with gas phase RH.

According to these assumptions, an aerosol particle consists of a number of concentric shells within the particle bulk in the Fi_PaD model. In this thesis, the number of shells is 400 and the resolution of a shell is around 10 nm for 4 μm radii of particle.

The Fi_PaD model is used to provide a forward simulation of the time-dependent size and response function

following the step change in the gas phase RH change [11,12]. A first guess for the water activity dependence of the diffusion coefficient is assumed. The diffusion coefficient of water in the mixture, D_w, is assumed to follow a Vignes form[11,12]

$$D_w(x,\alpha) = D_{w,w}^{x\alpha} \times D_{w,org}^{(1-x\alpha)}, \qquad (2)$$

where x is the mole fraction of water, $D_{w,w}$ is the known and limiting value of the diffusion coefficient of water in pure water, $D_{w,org}$ is diffusion coefficient of water in pure solute at infinite dilution of water.
α is often viewed as analogous to an activity coefficient, given by:

$$\ln \alpha = A(1-x)^3, \qquad (3)$$

where A is a temperature dependent parameter. using Fick's second law, the model simulates the concentration change of every shell in the particle by calculating the approximate diffusional mass flux.

All measurements are performed at room temperature (20°C). Thus, two fit parameters (A and $D_{w,org}$) are varied independently to achieve the best fit to the time-dependent size [12]. For the example shown in Figure 1(a), the best-fit water activity dependence of the diffusion coefficient of water in the mixture is shown in Figure 1(b).

Holographic Optical Tweezers

Holographic Optical Tweezers (HOT) are used to initiate the coalescence process between two aerosol particles and to, thereby, infer the particle viscosity at a particular gas phase RH/particle moisture content[2]. In order to catch multiple droplets, the optical additional components are required and are shown in Figure S5. The most significant is the inclusion of a Spatial Light Modulator (Holoeye SLM LC-R 2500, twisted nematic, liquid crystal on silicon) on the right side and a high bit-rate oscilloscope (LeCroy Wavesurfer 454) with photo detector (Thorlabs DET 110) on the left side, as shown in Figure S5. The trapping beam is a continuous wave Nd:YVO_4 laser at 532 nm (maximum output is 3W, Opus, Laser Quantum). After the laser output, the trapping beam is a vertically polarised by a half-wave plate, and it directly passes through a telescope which adjusts beam size. The SLM is used to split the beam. A beam expansion telescope and inverted microscope objective generate the optical traps to capture aerosol particles. Using the half-wave plate, the power division between the SLM and a beam dump can be controlled. In the trapping cell, multiple droplets can be held by the split beam. During experimental measurements, particle images are recorded by camera, and Raman spectra are recorded by the spectrograph. Light scattering patterns following the initiation of coalescence event are recorded by the photo detector.

Figure S5. A schematic representation of Holographic Optical Tweezers (HOT). The camera provides particle images, Raman spectrum is recorded by the spectrograph, and light scattering pattern is recorded by the photo detector.[2]

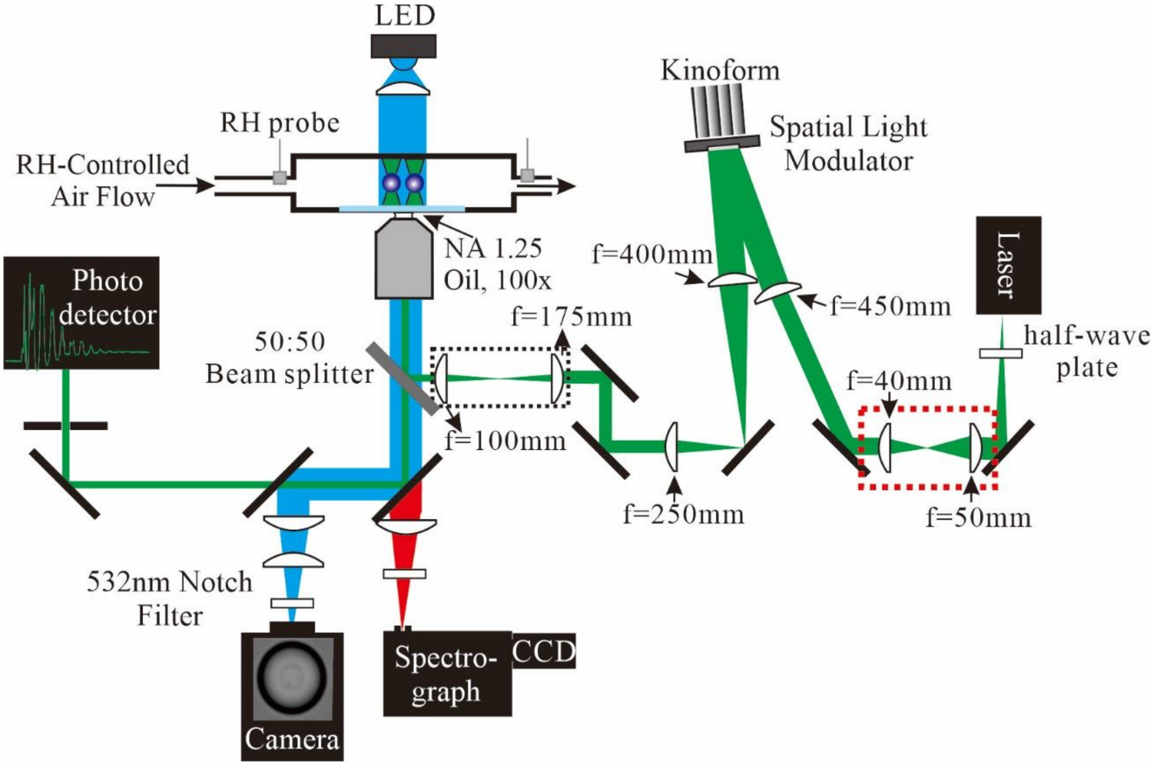

Viscosity of Saccharide Solutions Aerosol Particles

Previously, we have described the procedure for recording the viscosity of aerosols containing saccharides, alcohol, di- and tri-carboxylic acids, reporting viscosity measurements as a function of RH.[1,2,13] Briefly, pairs of aerosol particles of identical composition are captured in parallel optical traps formed using an holographic optical tweezers arrangement. The particles are conditioned at a fixed RH (in the range <5 to >90 % RH) for a period of time that can extend to hours. It has been shown that this allows sufficient time for the particles to achieve a moisture content in equilibrium with the gas phase RH and a uniform homogeneous composition/viscosity[2].

Coalescence is initiated by beam-steering, merging the pair of optical traps. At low viscosity (<10 Pa s), the particles may merge and relax in shape on a timescale less than 1 ms, a process that is monitored by measuring the relaxation in the back-scattered light from the optical trap. At higher viscosities, direct brightfield images can be recorded and the distortion in shape followed by determining the aspect ratio of the composite particle. The time-dependent decay in the aspect ratio can extend from milliseconds to >>10000 s, extending the viscosity range that can be measured from 1 Pa s up to 10^8 - 10^9 Pa s[2]. The viscosity is then inferred from the relaxation timescale, τ, assuming over-damped creeping fluid flow, the droplet radius and an estimate of the surface tension, σ:

$$\tau = \frac{\eta r}{\sigma} \qquad (4)$$

A comparison of the viscosity trends for saccharides from mono- to tri- structures is provided in Figure S6. There is a clear systematic trend toward higher viscosity as the composition progresses from a monosaccharide to a trisaccharide, depending on chemical structure, and molecular weight, with an order: glucose (180.16 g/mol) < sucrose (342.30 g/mol) < trehalose (342.296 g/mol) < maltose (342.30 g/mol) < raffinose (504.42 g/mol).

Figure S6. A comparison of viscosity of different systems: aqueous glucose (black), aqueous sucrose (red) aqueous trehalose (blue), aqueous maltose (pink), and aqueous raffinose (orange).[2] Figure is redrawn from Song et al.[2]

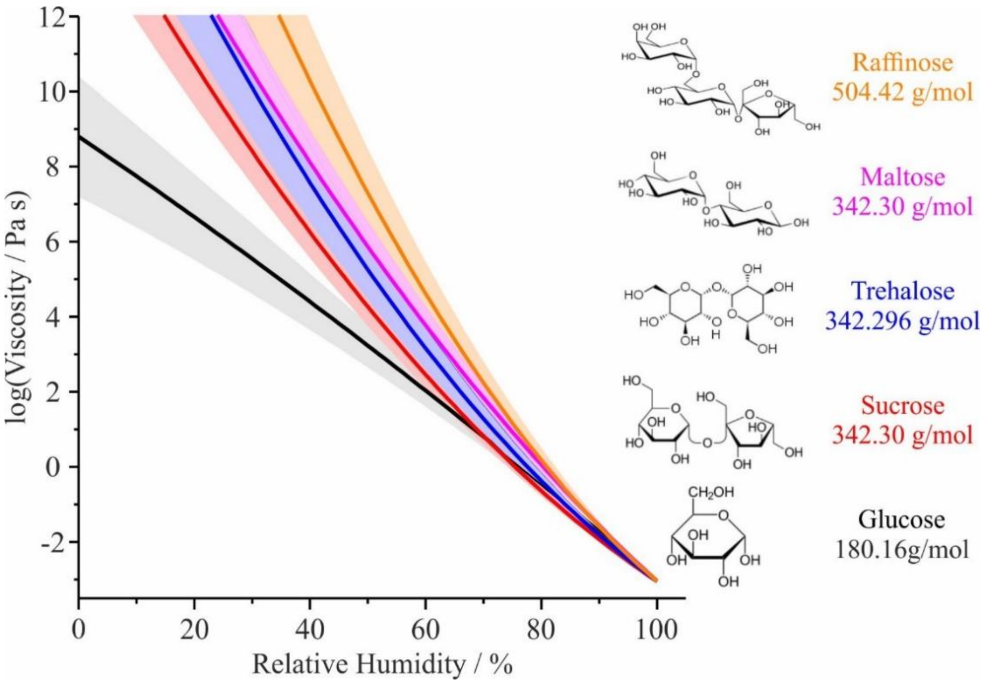

Molecular Dynamics Simulations

Force Fields

Water was represented by the TIP4P/2005[14] potential, due to its accuracy in reproducing the experimental phase and self diffusion characteristics. Sucrose was represented by a modified version of the GROMOS 54a7 force field[15], with an expanded range of atom types. Both the force field and the initial pdb all-atom coordinates were acquired from the automated topology builder (ATB) database[16] (further details on the generation[17] and validation[18] of coordinates, partial charges and force fields by ATB has been detailed extensively in the literature).

Initial coordinates were generated using the packmol program, which randomly places set numbers of molecules into three dimensional space, allowing tight control over the solute mole fractions in the generated simulation boxes, whilst not biasing the simulations to one area of configuration space. Constraints were inserted such that no two molecules were placed within 3Å of each other, and the input random seed was continuously replaced using the bash $RANDOM global variable.

The Lincs algorithm[19] was used to constrain all bonds, to an order of four in the constraint coupling matrix, with seven iterations in the final step. Electrostatic forces were calculated using the particle mesh Ewald summation[20], and Van der Waals interactions were provided by the twin range cut-offs method, both of which were truncated at 8Å. The update frequency was every 5 timesteps. The Verlet scheme[21] was used for neighbour searching across the periodic boundary conditions in three dimensions. Velocities were generated using a Maxwell-Boltzmann distribution at 300K, with the random seed continuously changed.

Equilibration

Water was represented by the TIP4P/2005[14] potential, due to its accuracy in reproducing the experimental phase and self diffusion characteristics. Sucrose was represented by a modified version of the GROMOS 54a7 force field [15], with an expanded range of atom types. Both the force field and the initial pdb all-atom coordinates were acquired from the automated topology builder (ATB) database [16] (further details on the generation [17] and validation [18] of coordinates, partial charges and force fields by ATB has been detailed elsewhere in the literature).

Initial coordinates were generated using the packmol program, which randomly places set numbers of molecules into three-dimensional space, allowing tight control over the solute mole fractions in the generated simulation boxes, whilst not biasing the simulations to one area of configuration space. Constraints were inserted such that no two molecules were placed within 3Å of each other, and the input random seed was continuously replaced using the bash $RANDOM global variable. The simulation box used in each case was a cubic unit cell of side length ~2.5 nm, periodic in all three dimensions.

The Lincs algorithm[19] was used to constrain all bonds, to an order of four in the constraint coupling matrix, with seven iterations in the final step. Electrostatic forces were calculated using the particle mesh Ewald summation[20], and Van der Waals interactions were provided by the twin range cut-offs method, both of which were truncated at 8Å. The update frequency was every 5 timesteps. The Verlet scheme[21] was used for neighbour searching across the periodic boundary conditions in three dimensions. Velocities were generated using a Maxwell-Boltzmann distribution at 300K, with the random seed continuously changed.

The starting coordinates produced by packmol are not suitable for MD simulations immediately. The configuration must be energy minimised using the steepest descent method to allow bonds and angles to satisfy

the constraints of the topology file. The minimisation was conducted with an initial step size of 0.001nm until the maximum force was below 50 $kJmol^{-1}nm^{-1}$. After this, approximately 500ps of equilibration was conducted with the standard GROMACS md integrator and thermodynamic ensemble produced as described in the main text.

Mean squared displacement data collection

Initial coordinates were generated from the output frame of the equilibration trajectory within the regime where the total energy was stable. During the MD integration, the system was propagated in the NpT ensemble at 300K and 1 atmosphere, using a velocity rescaling thermostat [22] and the Parrinello-Rahman barostat [23], respectively. Initial velocities were randomly generated to satisfy a Maxwell-Boltzmann distribution at 300 K in each case. All simulations described were conducted using GPU acceleration, with the MD package GROMACS (version 5.0.6)[24], running on the Blue Crystal 3 high performance computing cluster at the University of Bristol.

Due to the extremely kinetically limited state of the aqueous-sucrose system, it is necessary to simulate dynamics for very long periods of time, relative to, for example, the timescale of molecular vibration or rotation. This maximises the probability that the initial conditions are overcome, and that the constituent molecules decorrelated from their initial conditions as the simulation proceeds. In each of the trajectories of caged water, dynamics were computed for 1 μs, and it was found that, when averaged over all nine trajectories, the mean squared displacements of the water molecules converged to a diffusive dependence on simulation time, *t*, (Figure S7).

$$\langle r^2 \rangle = 2D_{w,org}t \qquad (5)$$

The net displacement achieved by a random walk in three-dimensions will be less than the path length taken between the initial and final positions, a consequence of the fact that the translational motion of water through such a lattice is not Brownian. All nine trajectories contain segments during which the water is travelling perpendicularly, or even backwards, relative to its net displacement. It may be the case that the decorrelation of the water velocity is limited by the rearrangement of the sucrose, as well as the thermodynamic barrier that must be overcome to hop to the nearest available cavity, rather than the dynamics of a more typical solvation shell in an aqueous environment. Therefore, it is desirable to calculate the magnitude of the displacement to the current position, $|\langle r^2 \rangle|$, at every timestep, rather than cumulatively sum the path length that takes into account every intermediate 'jump'. Additionally, removal from the calculation of the centre of mass motion of the water molecule ensures that any atomically resolved vibrational motion does not contribute to the calculation of net displacement. The value stated in the manuscript and illustrated in Fig 1c was calculated under these conditions.

Figure S7: Single particle mean squared displacements of nine trajectories (purple), along with best fit of the mean path (red) to equation (1) (yellow, including constant at t=0).

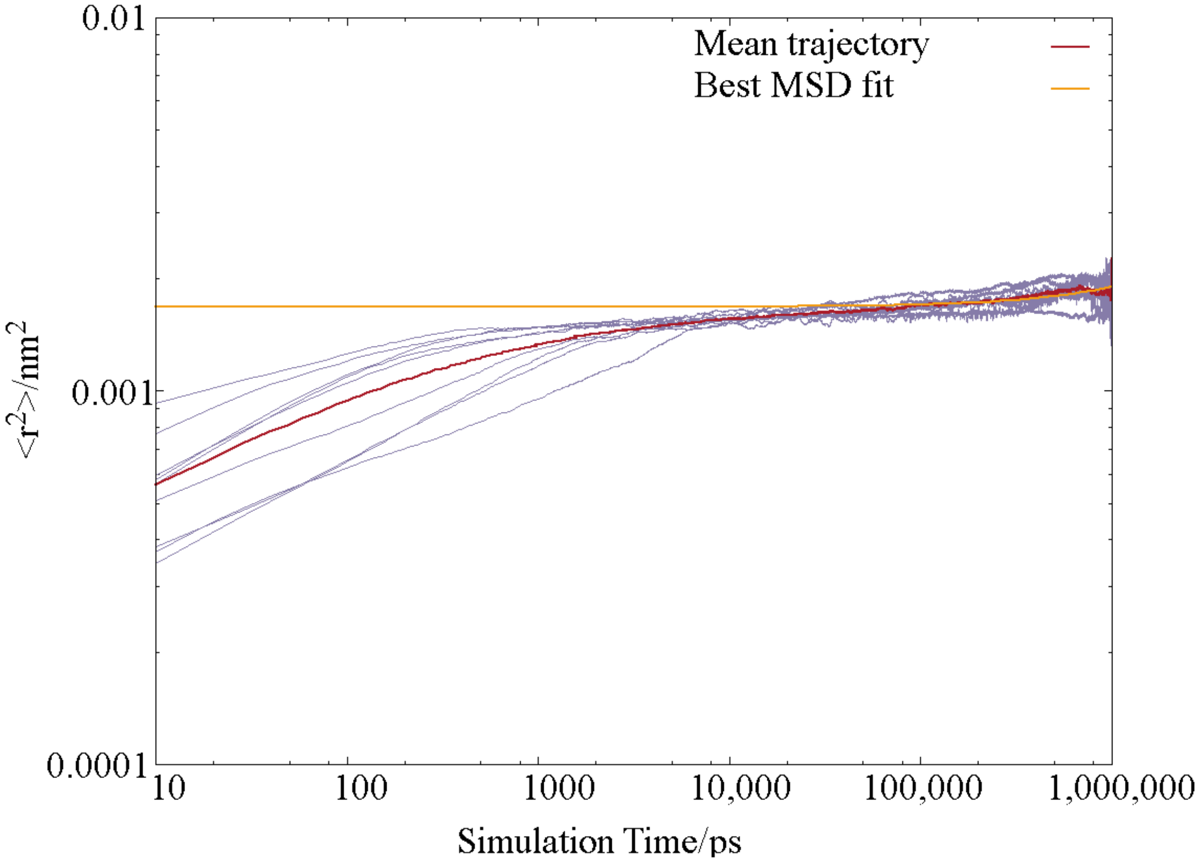

These MD simulations were not designed to capture diffusion against a chemical potential gradient, which is what is induced and probed during the optical tweezers measurements reported above. Instead we wished to treat the water as a tracer particle moving stochastically through the matrix, subject to a small localization uncertainty arising from motion between adjacent frames.[25] This is the origin of the offset at t=0 in the figure. The per simulation D values are presented in table S2.

A literature review was conducted to investigate whether any corrections or different functional forms of mean squared displacement needed to be fit. A recent publication [26] by Alcazar-Cano and Delgado-Buscalioni has suggested that in systems where the diffusing 'tracer' particles are trapped in the manner described here, and cannot freely move through channels, it may be more appropriate to fit $\langle r^2 \rangle$ to a subdiffusive dependence, namely,

$$\langle r^2 \rangle \sim D_{w,org} t^{\alpha} \qquad (6)$$

where $\alpha \to 0$ as the proportion of particles that are trapped approaches 100%. Similar physics was described by Zwanzig earlier, in 1988,[27] although it was incorporated into the mathematics by correcting *D,* rather than

t. Specifically, Zwanzig considered a so-called 'rough potential' where the particle under consideration must traverse a landscape of many nearly degenerate local minima. In that case, the observed D is smaller than the effective D by a factor ϵ that accounts for the 'roughness' of the free energy landscape.

$$D_{w,org} = D_{effective} \exp\left(-\frac{\epsilon}{kT}\right)^{2} \qquad (7)$$

These phenomena are often observed in conjunction with stretched exponential relaxation in sugar solutions,[28,29] which the radius curves in Figure 1(a) also exhibit.

Calculating Free volume

Three repeat trajectories of 10 ns length were conducted for glucose and raffinose, once again generated with randomly placed and oriented molecules via packmol and containing a single water. To determine the sucrose packing efficiency, truncated trajectories 10 ns long were extracted from the microsecond trajectories and subject to the same analysis, using the GROMACS 'free-volume' program, which attempts to insert 'dummy' probe particles into the box. The free volume calculated is the total volume of the successful insertions.

Markov state modelling

The goal of the Markov state modelling was to identify regions of the MD simulations in local equilibrium, to identify cavities, and calculate their associated hopping and relaxation timescales. Eight MD simulations were partitioned into 1 ns time slices and the position of the water center of mass was clustered into 100 discrete states using the KMeans clustering algorithm. The KMeans clustering and all subsequent calculations were performed using the open source software, PyEMMA[30] .

The discrete trajectories were screened to see whether they were in local equilibrium with the following procedure:

1. A Markov state model (MSM) with a lag time (τ) of 10ps was constructed for each time slice. The lag time was chosen so as to reveal details of potential metastability[31]. To determine the value of τ, we carried out a sensitivity analysis, varying τ until we observed convergence in the eigenvalues. See figure S10 panel (b & f).
2. This MSM was coarse grained into a k-state Hidden Markov Model (HMM) if the largest gap in successive implied timescales of the MSM $\left({t_k}/{t_{k+1}}\right)$ was greater than 1.5.
3. If the HMM had:
    a. an absorbing state (a 1 on the diagonal of the transition matrix);
    b. a metastable state, i, with stationary distribution (π_i) probability approximately zero ($\pi_i < 10^{-8}$);

c. negative eigenvalues;

it was classified as not being in local equilibrium. The conditions (a – c) are indicative of not being in equilibrium because of the enforcement of the detailed balance conditions when estimating the HMM transition matrix[32].

4. The time-slices identified as being in local equilibrium were used to construct a Bayesian HMM in order to estimate errors of the hopping timescales.

Out of the 8000 time slices analysed 947 (11.8%) were positively identified as being in local equilibrium. Figure S10 demonstrate this classification algorithm for two time slices of trajectory 3. The first time slice (0 – 1ns, panels a – d) was classified as not being in local equilibrium, the second time slice (22 – 23n, panels e – h) was classified as being in local equilibrium.

Figure S8. Position-position autocorrelation function of a single sucrose molecule within the simulation, showing very little motion in the region 0.1-1000 ps. This plot suggests that it is reasonable cluster the data (to determine cavities, as in Figure 4) in increments of 1 ns. It is also reasonable to initialise frozen sucrose simulations after intervals of 1 ns (Figure 3, panels b-d).

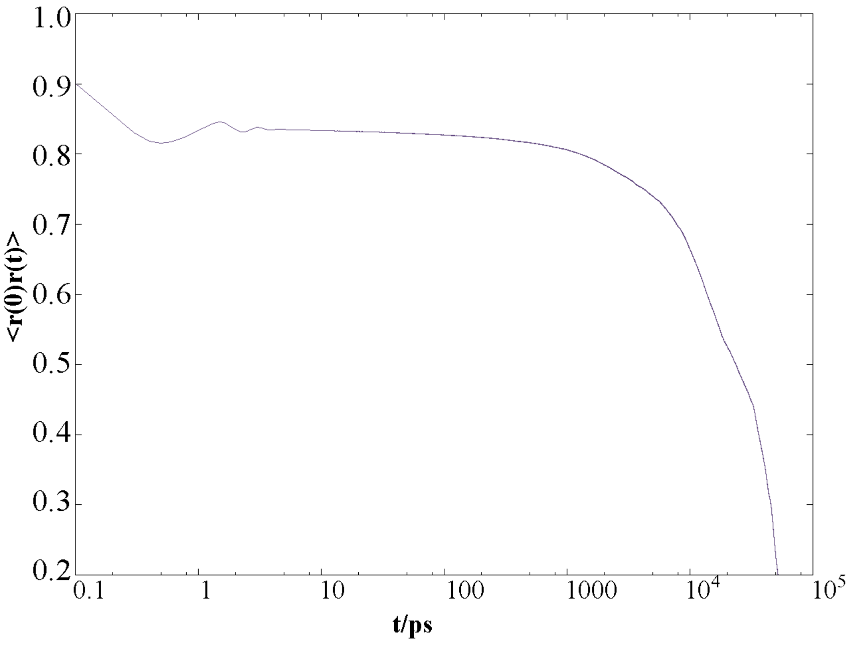

Figure S9. Histogram of barrier heights for eight trajectories, calculated in 1 ns intervals. Free energies are calculated by converting the hopping timescales (t) of the hidden Markov models to free energies (ΔG) using $\Delta G = RT \ln\left({k_b T \cdot t}/{h}\right)$.

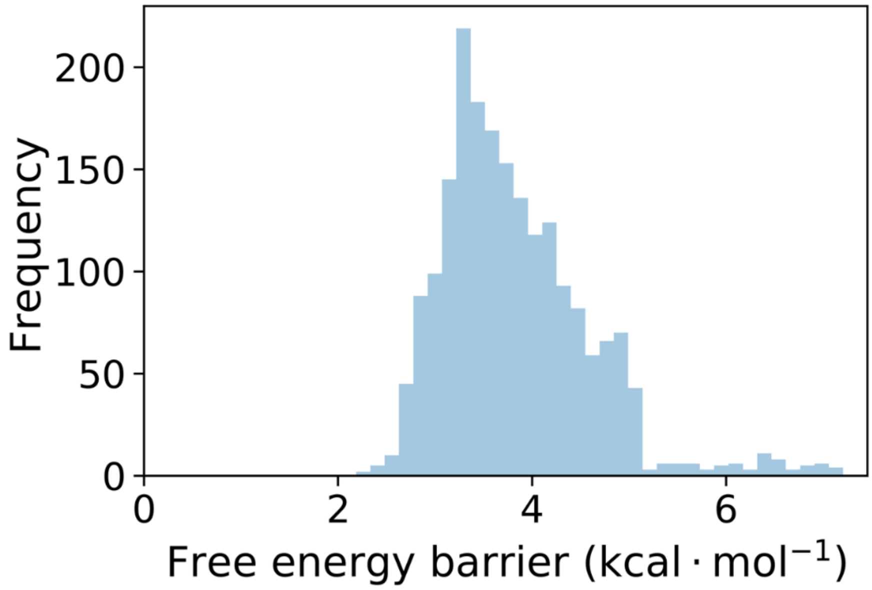

Figure S10. Classification of two time slices from trajectory 3: 0 – 1ns (panels a – d, non-equilibrium time slice) and 22 – 23ns (panels e – h, local equilibrium time slice). Panel (a) and (e) show the x, y, and z coordinates of the center of mass of the water molecule. Panel (b) and (f) show the sensitivity analysis to determine the lag time τ: the implied timescales, (t, vertical axis) vs the MSM lag, (τ, horizontal axis). The black vertical line is place at 10ps is the minimum time at which the implied timescales show convergence. Panels (c) and (g) show the stationary distribution. In panel (c) state 1 has negligible probability classifying this time slice as not being in equilibrium. Panels (d) and (h) show the attempted assignment of the discrete trajectory into metastable states. The non-equilibrium time slice in panel (d) shows the large portion of unclassified states.

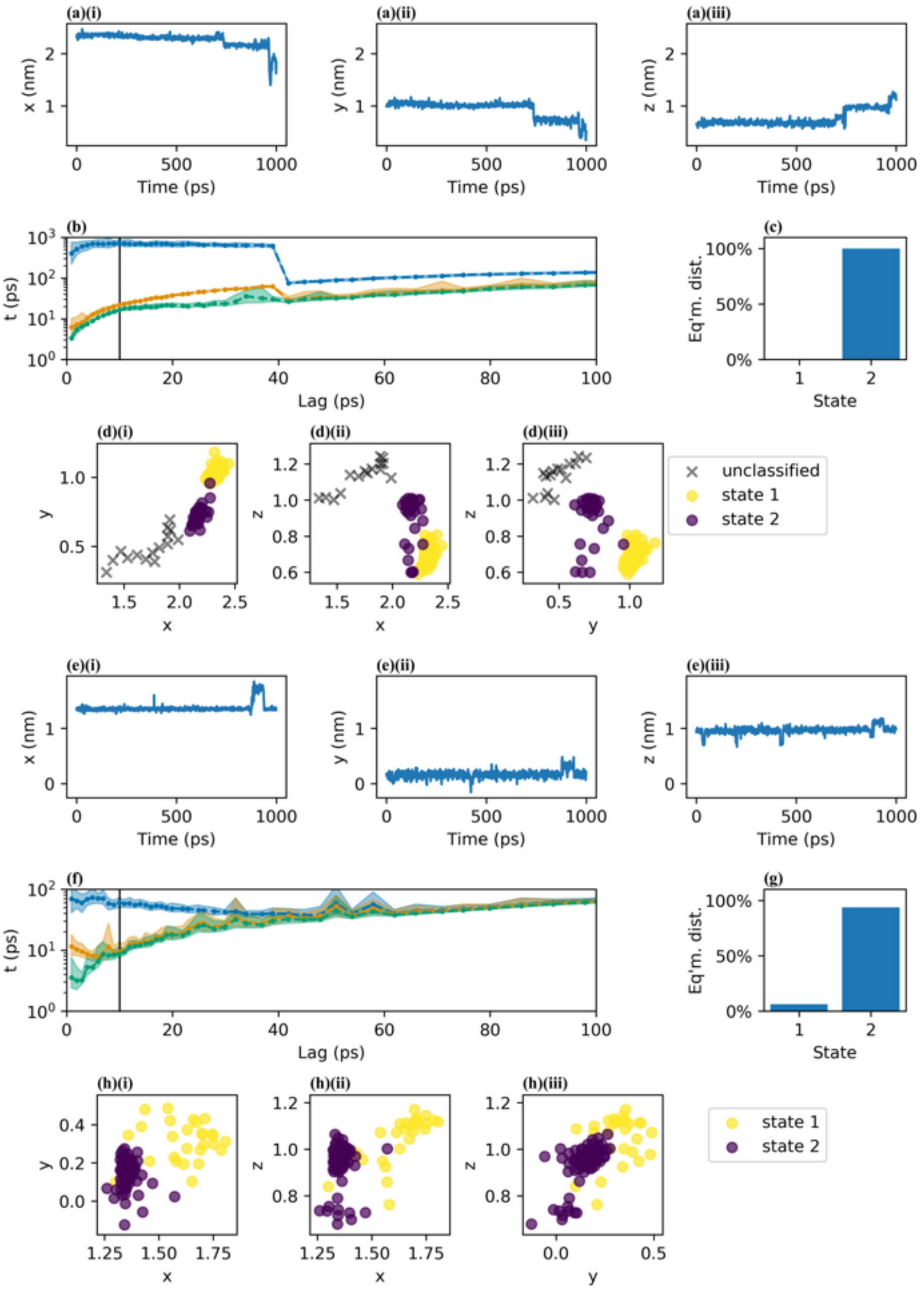

The comparison of diffusion coefficient of aqueous-sucrose system

For investigating diffusion in aerosol systems, sucrose has become the representative organic system. A comparison of diffusion curves of sucrose as a function of water activity reported by different groups is shown in Figure S11. The diffusion constants of water in aqueous-sucrose system in Figure S11 are measured and simulated from different measurements strategies using AOT with Fi_Pad model, EDB with ETH model, and isotopic exchange. These three parameterisations show good agreement above a water activity 0.35. The diffusion constants of water at 0.2 water activity by Price et al.[33] and this research differ by one order of magnitude, as previously discussed.[12]

Figure S11. The diffusion curve of water in a sucrose system as a function of water activity. The green line is from Zobrist *et al.*[34], the blue line from Price *et al.*[33], and the red line is the experimental data which is simulated by Fi_PaD model.

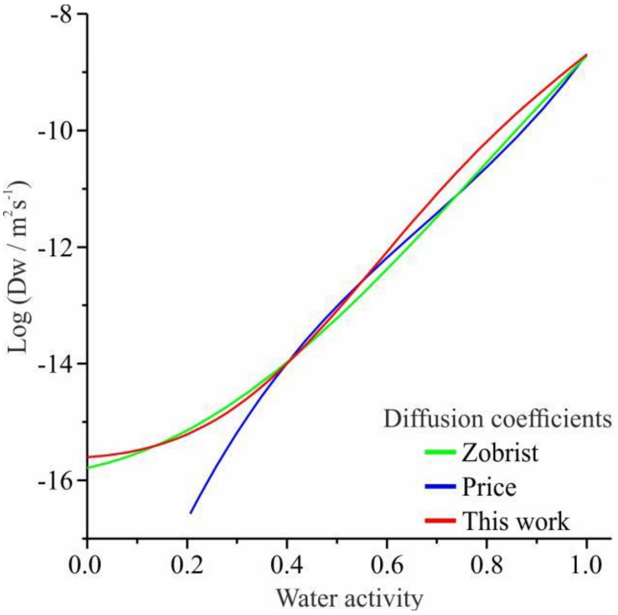

Table S1. The Characteristic timescale of water transport determined experimentally for six binary mixtures. This table provides all data points for water transport kinetics in Figure S2. Particle size is direct measurement data in AOT and fit by LARA. The characteristic timescale is fit by KWW function. Error representing a variation of $\beta \pm 0.1$.

**Aqueous-Glucose system**			**Aqueous-Maltose system**		
RH transition	Particle size / nm	The Characteristic timescale / s	RH transition	Particle size / nm	The Characteristic timescale / s
50% - 30%	3707	27	50% - 30%	368	3721
	3692	27		514	4114
	3871	29		534	4329
	4861	33		549	5131
50% - 20%	3707	49	30% - 5%	3664	3289
	3692	51		2624	3446
	4861	92		4062	3745
30% - 5%	3377	860		4845	4197
	4053	1007		3996	4355
	3871	1009		5755	4662
	4412	1084		1641	4130
	4988	1289	30% - 50%	436	3265
	5450	1326		423	3268
30% - 50%	3707	49		441	4101
	3692	51		597	5012
	4861	63		617	5430
20% - 50%	3707	95	5% - 30%	960	3446
5% - 30%	3377	140		1490	4197
	4412	190		1250	4662
	5450	276		1230	4145
				578	4130
**Aqueous-Raffinose system**			**Aqueous-Sucrose system**		
RH transition	Particle size / nm	The Characteristic timescale / s	RH transition	Particle size / nm	The Characteristic timescale / s
50-30	3332	188	50-10	3052	397
--	3414	221	--	3307	535
--	3967	250	--	4092	883
--	4110	265	--	4013	1019
--	5005	298	--	4954	1076
--	4924	313	50-20	3796	244

50-20	3260	153	--	4189	363
--	3332	254	--	4783	540
--	3410	288	--	4942	559
--	3966	284	50-30	3682	48
--	3606	260	--	4016	59
--	3779	300	--	4894	65
--	4105	320	--	4923	60
--	5008	390	--	5745	85
50-10	3260	178	30-20	3068	668
--	3410	352	--	3123	847
--	3719	700	--	3651	1502
--	5008	1010	--	4382	1748
30-10	3551	1210	--	4758	2274
--	5876	1906	30-10	3297	1243
30-5	3551	895	--	3654	1786
--	3735	995	--	3655	1901
--	3952	1447	--	3652	1924
--	4343	1578	--	4020	2141
--	5895	2101	--	4017	2191
30-50	3332	482	--	4308	2277
--	3414	495	--	3891	2379
--	3967	547	--	5727	3582
--	4110	738	--	4824	4374
--	4924	617	30-5	3246	1321
--	5005	730	--	3657	1804
20-50	3262	432	--	3809	2035
--	3332	351	--	3754	2061
--	3414	440	--	4987	3584
--	3606	476	--	5163	3738
--	3967	399	--	5266	3763
--	5005	516	30-50	3426	57
5-30	3551	610	--	3829	77
--	3735	514	--	4042	79
--	3952	852	--	4962	112
--	4343	646	5-30	3252	1776
--	5895	926		3645	2059
				3563	2395
--	--	--		3633	2566
				3983	2763

				4716	3056
				4912	3294
				5912	4112
**Aqueous-Trehalose system**			**Aqueous-Levoglucosan system**		
RH transition	Particle size / nm	The Characteristic timescale / s	RH transition	Particle size / nm	The Characteristic timescale / s
50-30	3265	98	50-30	3590	35
--	3268	101		3588	40
--	4101	157		3746	32
--	5430	231		4432	43
--	5012	204		4463	47
50-20	3265	221		5448	53
--	3268	194	30-50	3588	81
--	4101	184		3746	52
--	5012	244		4432	117
--	5430	233		4463	86
30-5	3090	1019		5448	76
--	3190	1172			
--	3285	1155			
--	3310	1184			
--	4040	1342			
--	4825	1479			
--	5168	1559			
--	5118	1444			
30-50	3265	436			
--	3268	423			
--	4101	441			
--	5430	617			
--	5012	597			
20-50	3265	181			
5-30	4040	726			
--	3190	515			
--	3090	592			
--	5168	846			
--	3853	589			
--	4897	719			
--	5118	981			
	3310	596			

Table S2. The best fit diffusion coefficients to the nine 1μs trajectories (purple lines in Figure S7) and associated uncertainties.

Trajectory #	D/m^2s^{-1}	Uncertainty/m^2s^{-1}
1	6.454E-17	9.34E-17
2	4.848E-17	1.755E-16
3	6.075E-17	1.444E-16
4	8.922E-18	3.448E-17
5	1.004E-18	1.88E-16
6	8.775E-17	2.218E-17
7	3.541E-17	8.571E-17
8	3.829E-17	1.61E-17
9	7.269E-17	5.83E-17

Table S3. The best fit diffusion coefficients of six binary systems in Figure 1(c).

System	Parameterization ($D_w(a_w)/m^2s^{-1}$)
Aqueous-Levoglucosan	$\log (D_w(a_w)) = -14.091 + (2.192 \times a_w) + (-21.269 \times (a_w)^2) + (81.025 \times (a_w)^3) + (-84.660 \times (a_w)^4) + (28.081 \times (a_w)^5)$
Aqueous-glucose	$\log (D_w(a_w)) = -15.047 + (0.963 \times a_w) + (-0.186 \times (a_w)^2) + (34.825 \times (a_w)^3) + (-47.724 \times (a_w)^4) + (18.472 \times (a_w)^5)$
Aqueous-sucrose	$\log (D_w(a_w)) = -15.613 + (1.262 \times a_w) + (-3.476 \times (a_w)^2) + (46.468 \times (a_w)^3) + (-60.030 \times (a_w)^4) + (22.691 \times (a_w)^5)$
Aqueous-Trehalose	$\log (D_w(a_w)) = -15.503 + (1.061 \times a_w) + (0.642 \times (a_w)^2) + (34.712 \times (a_w)^3) + (-48.590 \times (a_w)^4) + (18.981 \times (a_w)^5)$
Aqueous-Maltose	$\log (D_w(a_w)) = -15.857 + (1.269 \times a_w) + (-6.922 \times (a_w)^2) + (58.525\times (a_w)^3) + (-72.854 \times (a_w)^4) + (27.141 \times (a_w)^5)$
Aqueous-Raffinose	$\log (D_w(a_w)) = -15.393 + (1.113 \times a_w) + (2.180 \times (a_w)^2) + (29.194 \times (a_w)^3) + (-42.838 \times (a_w)^4) + (17.051 \times (a_w)^5)$

References

(1) Power, R. M.; Reid, J. P. Probing the Micro-Rheological Properties of Aerosol Particles Using Optical Tweezers. *Reports Prog. Phys.* **2014**, *77* (7), 074601. https://doi.org/10.1088/0034-4885/77/7/074601.

(2) Song, Y. C.; Haddrell, A. E.; Bzdek, B. R.; Reid, J. P.; Bannan, T.; Topping, D. O.; Percival, C.; Cai, C. Measurements and Predictions of Binary Component Aerosol Particle Viscosity. *J. Phys. Chem. A* **2016**, *120* (41), 8123–8137. https://doi.org/10.1021/acs.jpca.6b07835.

(3) Preston, T. C.; Reid, J. P. Accurate and Efficient Determination of the Radius, Refractive Index, and Dispersion of Weakly Absorbing Spherical Particle Using Whispering Gallery Modes. *J. Opt. Soc. Am. B* **2013**, *30* (8), 2113–2122. https://doi.org/10.1364/JOSAB.30.002113.

(4) Reid, J. P.; Meresman, H.; Mitchem, L.; Symes†, R. Spectroscopic Studies of the Size and Composition of Single Aerosol Droplets. *Int. Rev. Phys. Chem.* **2007**, *26* (1), 139–192. https://doi.org/10.1080/01442350601081899.

(5) Reid, J. P.; Mitchem, L. Laser Probing of Single-Aerosol Droplet Dynamics. *Annu. Rev. Phys. Chem.* **2006**, *57*, 245–271. https://doi.org/10.1146/annurev.physchem.57.032905.104621.

(6) Rickards, A. M. J.; Song, Y. C.; Miles, R. E. H.; Preston, T. C.; Reid, J. P. Variabilities and Uncertainties in Characterising Water Transport Kinetics in Glassy and Ultraviscous Aerosol. *Phys. Chem. Chem. Phys.* **2015**, *17* (15), 10059–10073. https://doi.org/10.1039/c4cp05383d.

(7) Tong, H.-J.; Reid, J. P.; Bones, D. L.; Luo, B. P.; Krieger, U. K. Measurements of the Timescales for the Mass Transfer of Water in Glassy Aerosol at Low Relative Humidity and Ambient Temperature. *Atmos. Chem. Phys.* **2011**, *11* (10), 4739–4754. https://doi.org/10.5194/acp-11-4739-2011.

(8) Chen, T.; Fowler, a; Toner, M. Literature Review: Supplemented Phase Diagram of the Trehalose-Water Binary Mixture. *Cryobiology* **2000**, *40* (3), 277–282. https://doi.org/10.1006/cryo.2000.2244.

(9) Foster, K. D.; Bronlund, J. E.; Paterson, A. H. J. (Tony). Glass Transition Related Cohesion of Amorphous Sugar Powders. *J. Food Eng.* **2006**, *77* (4), 997–1006. https://doi.org/10.1016/j.jfoodeng.2005.08.028.

(10) Lu, J. W.; Rickards, A. M. J.; Walker, J. S.; Knox, K. J.; Miles, R. E. H.; Reid, J. P.; Signorell, R. Timescales of Water Transport in Viscous Aerosol: Measurements on Sub-Micron Particles and Dependence on Conditioning History. *Phys. Chem. Chem. Phys.* **2014**, *16* (21), 9819–9830. https://doi.org/10.1039/C3CP54233E.

(11) O’Meara, S.; Topping, D. .; McFiggans, G. The Rate of Equilibration of Viscous Aerosol Particles. *Atmos. Chem. Phys.* **2016**, *16*, 5299–5313. https://doi.org/10.5194/acp-2015-1019.

(12) Ingram, S.; Cai, C.; Song, Y. C.; Glowacki, D. R.; Topping, D. O.; O’Meara, S.; Reid, J. P. Characterising the Evaporation Kinetics of Water and Semi-Volatile Organic Compounds from Viscous Multicomponent Organic Aerosol Particles. *Phys. Chem. Chem. Phys.* **2017**, *19* (47), 31634–31646. https://doi.org/10.1039/c7cp05172g.

(13) Power, R. M.; Simpson, S. H.; Reid, J. P.; Hudson, A. J. The Transition from Liquid to Solid-Like Behaviour in Ultrahigh Viscosity Aerosol Particles. *Chem. Sci.* **2013**, *4* (6), 2597–2604. https://doi.org/10.1039/c3sc50682g.

(14) Abascal, J. L. F.; Vega, C. A General Purpose Model for the Condensed Phases of Water: TIP4P/2005. *J. Chem. Phys.* **2005**, *123* (23), 234505. https://doi.org/10.1063/1.2121687.

(15) Oostenbrink, C.; Villa, A.; Mark, A. E.; Van Gunsteren, W. F. A Biomolecular Force Field Based on the Free Enthalpy of Hydration and Solvation: The GROMOS Force-Field Parameter Sets 53A5 and 53A6. *J. Comput. Chem.* **2004**, *25* (13), 1656–1676. https://doi.org/10.1002/jcc.20090.

(16) Koziara, K. B.; Stroet, M.; Malde, A. K.; Mark, A. E. Testing and Validation of the Automated Topology Builder (ATB) Version 2.0: Prediction of Hydration Free Enthalpies. *J. Comput. Aided. Mol. Des.* **2014**, *28* (3), 221–233. https://doi.org/10.1007/s10822-014-9713-7.

(17) Malde, A. K.; Zuo, L.; Breeze, M.; Stroet, M.; Poger, D.; Nair, P. C.; Oostenbrink, C.; Mark, A. E. An Automated Force Field Topology Builder (ATB) and Repository: Version 1.0. *J. Chem. Theory Comput.* **2011**, *7* (12), 4026–4037. https://doi.org/10.1021/ct200196m.

(18) Schmid, N.; Eichenberger, A. P.; Choutko, A.; Riniker, S.; Winger, M.; Mark, A. E.; van Gunsteren, W. F. Definition and Testing of the GROMOS Force-Field Versions 54A7 and 54B7. *Eur. Biophys. J.* **2011**, *40* (7), 843–856. https://doi.org/10.1007/s00249-011-0700-9.

(19) Hess, B.; Bekker, H.; Berendsen, H. J. C.; Fraaije, J. G. E. M. LINCS: A Linear Constraint Solver for Molecular Simulations. *J. Comput. Chem.* **1997**, *18* (12), 1463–1472. https://doi.org/10.1002/(SICI)1096-987X(199709)18:12<1463::AID-JCC4>3.0.CO;2-H.

(20) Essmann, U.; Perera, L.; Berkowitz, M. L.; Darden, T.; Lee, H.; Pedersen, L. G. A Smooth Particle Mesh Ewald Method. *J. Chem. Phys.* **1995**, *103* (19), 8577. https://doi.org/10.1063/1.470117.

(21) Páll, S.; Hess, B. A Flexible Algorithm for Calculating Pair Interactions on SIMD Architectures. *Comput. Phys. Commun.* **2013**, *184* (12), 2641–2650. https://doi.org/10.1016/j.cpc.2013.06.003.

(22) Bussi, G.; Donadio, D.; Parrinello, M. Canonical Sampling through Velocity Rescaling. *J. Chem. Phys.* **2007**, *126* (1), 014101. https://doi.org/10.1063/1.2408420.

(23) Parrinello, M.; Rahman, A. Polymorphic Transitions in Single Crystals: A New Molecular Dynamics Method. *J. Appl. Phys.* **1981**, *52* (12), 7182–7190. https://doi.org/10.1063/1.328693.

(24) Abraham, M. J.; Murtola, T.; Schulz, R.; Páll, S.; Smith, J. C.; Hess, B.; Lindahl, E. GROMACS: High Performance Molecular Simulations through Multi-Level Parallelism from Laptops to Supercomputers. *SoftwareX* **2015**, *1–2*, 19–25. https://doi.org/10.1016/j.softx.2015.06.001.

(25) Michalet, X. Mean Square Displacement Analysis of Single-Particle Trajectories with Localization Error: Brownian Motion in an Isotropic Medium. *Phys. Rev. E* **2010**, *82* (4), 041914. https://doi.org/10.1103/PhysRevE.82.041914.

(26) Alcázar-Cano, N.; Delgado-Buscalioni, R. A General Phenomenological Relation for the Subdiffusive Exponent of Anomalous Diffusion in Disordered Media. *Soft Matter* **2018**, *14* (48), 9937–9949. https://doi.org/10.1039/C8SM01961D.

(27) Zwanzig, R. Diffusion in a Rough Potential. *Proc. Natl. Acad. Sci.* **1988**, *85* (7), 2029–2030. https://doi.org/10.1073/pnas.85.7.2029.

(28) Meste, M. Le; Champion, D.; Roudaut, G.; Blond, G.; Simatos, D. Glass Transition and Food Technology: A Critical Appraisal. *J. Food Sci.* **2002**, *67* (7), 2444–2458. https://doi.org/10.1111/j.1365-2621.2002.tb08758.x.

(29) Liu, Y.; Bhandari, B.; Zhou, W. Glass Transition and Enthalpy Relaxation of Amorphous Food Saccharides: A Review. *J. Agric. Food Chem.* **2006**, *54* (16), 5701–5717. https://doi.org/10.1021/jf060188r.

(30) Scherer, M. K.; Trendelkamp-Schroer, B.; Paul, F.; Pérez-Hernández, G.; Hoffmann, M.; Plattner, N.; Wehmeyer, C.; Prinz, J.-H.; Noé, F. PyEMMA 2: A Software Package for Estimation, Validation, and Analysis of Markov Models. *J. Chem. Theory Comput.* **2015**, *11* (11), 5525–5542. https://doi.org/10.1021/acs.jctc.5b00743.

(31) Prinz, J.-H.; Wu, H.; Sarich, M.; Keller, B.; Senne, M.; Held, M.; Chodera, J. D.; Schütte, C.; Noé, F. Markov Models of Molecular Kinetics: Generation and Validation. *J. Chem. Phys.* **2011**, *134* (17), 174105. https://doi.org/10.1063/1.3565032.

(32) Noé, F.; Wu, H.; Prinz, J.-H.; Plattner, N. Projected and Hidden Markov Models for Calculating Kinetics and Metastable States of Complex Molecules. *J. Chem. Phys.* **2013**, *139* (18), 184114. https://doi.org/10.1063/1.4828816.

(33) Price, H. C.; Murray, B. J.; Mattsson, J.; O'Sullivan, D.; Wilson, T. W.; Baustian, K. J.; Benning, L. G. Quantifying Water Diffusion in High-Viscosity and Glassy Aqueous Solutions Using a Raman Isotope Tracer Method. *Atmos. Chem. Phys.* **2014**, *14* (8), 3817–3830. https://doi.org/10.5194/acp-14-3817-2014.

(34) Zobrist, B.; Soonsin, V.; Luo, B. P.; Krieger, U. K.; Marcolli, C.; Peter, T.; Koop, T. Ultra-Slow Water Diffusion in Aqueous Sucrose Glasses. *Phys. Chem. Chem. Phys.* **2011**, *13* (8), 3514–3526. https://doi.org/10.1039/c0cp01273d.
